# Comparison of Microbiomes from Different Niches of Upper and Lower Airways in Children and Adolescents with Cystic Fibrosis

**DOI:** 10.1371/journal.pone.0116029

**Published:** 2015-01-28

**Authors:** Sébastien Boutin, Simon Y. Graeber, Michael Weitnauer, Jessica Panitz, Mirjam Stahl, Diana Clausznitzer, Lars Kaderali, Gisli Einarsson, Michael M. Tunney, J. Stuart Elborn, Marcus A. Mall, Alexander H. Dalpke

**Affiliations:** 1 Dept. of Infectious Diseases—Medical Microbiology and Hygiene, University Hospital Heidelberg, Heidelberg, Germany; 2 Department of Translational Pulmonology, University Hospital Heidelberg, Heidelberg, Germany; 3 Div. of Pediatric Pulmonology & Allergology and Cystic Fibrosis Center, Dept. of Pediatrics, University Hospital Heidelberg, Heidelberg, Germany; 4 Translational Lung Research Center Heidelberg (TLRC), Member of the German Center for Lung Research (DZL), Heidelberg, Germany; 5 Institute for Medical Informatics and Biometry, Technical University Dresden, Dresden, Germany; 6 CF & Airways Microbiology Group, Queen’s University Belfast, Belfast, United Kingdom; 7 School of Pharmacy, Queen’s University Belfast, Belfast, United Kingdom; 8 Centre for Infection & Immunity, School of Medicine, Dentistry & Biomedical Science, Queen’s University Belfast, Belfast, United Kingdom; Ghent University, BELGIUM

## Abstract

Changes in the airway microbiome may be important in the pathophysiology of chronic lung disease in patients with cystic fibrosis. However, little is known about the microbiome in early cystic fibrosis lung disease and the relationship between the microbiomes from different niches in the upper and lower airways. Therefore, in this cross-sectional study, we examined the relationship between the microbiome in the upper (nose and throat) and lower (sputum) airways from children with cystic fibrosis using next generation sequencing. Our results demonstrate a significant difference in both α and β-diversity between the nose and the two other sampling sites. The nasal microbiome was characterized by a polymicrobial community while the throat and sputum communities were less diverse and dominated by a few operational taxonomic units. Moreover, sputum and throat microbiomes were closely related especially in patients with clinically stable lung disease. There was a high inter-individual variability in sputum samples primarily due to a decrease in evenness linked to increased abundance of potential respiratory pathogens such as *Pseudomonas aeruginosa*. Patients with chronic *Pseudomonas aeruginosa* infection exhibited a less diverse sputum microbiome. A high concordance was found between pediatric and adult sputum microbiomes except that *Burkholderia* was only observed in the adult cohort. These results indicate that an adult-like lower airways microbiome is established early in life and that throat swabs may be a good surrogate in clinically stable children with cystic fibrosis without chronic *Pseudomonas aeruginosa* infection in whom sputum sampling is often not feasible.

## Introduction

Cystic fibrosis (CF) is caused by mutations in the cystic fibrosis transmembrane conductance regulator (*CFTR*) gene and remains one of the most frequent lethal hereditary diseases in Caucasian populations [1]. Although CF affects various organs, over 85% of the morbidity and mortality of the disease are attributed to an early onset lung disease that is characterized by airway mucus obstruction and intermittent to chronic infection with characteristic pathogens such as *Staphylococcus aureus*, *Haemophilus influenza*, *Pseudomonas aeruginosa* and *Burkholderia cepacia* complex. This results in and chronic neutrophilic inflammation leading to bronchiectasis and progressive lung damage [2–4].

The airways are composed of different interconnected niches, namely the nasal sinuses, the throat and the conducting airways. Bacterial colonization in the nose and throat is considered normal and these niches harbor a complex microbiome [5,6]. Until recently, the lower airways were considered sterile and detection of pathogens indicative of bacterial infection [7,8]. However, culture independent methods have shown that the lungs are not sterile even in healthy people [9,10]. In CF patients, the lungs are colonized by a polymicrobial community containing a diverse array of organisms including potential respiratory pathogens (PRPs) [9,11]. The dynamics of the microbiome in the lower airways evolves from an initial diversification in younger CF patients followed by a reduction and selection of PRPs frequently associated with a decline in pulmonary function in older patient populations [12]. A similar shift from a polymicrobial community to a pathogen centered community linked to disease has already been described in both the gut and oral cavity [13–15].

The lungs are the target of colonization by bacteria from the environment via the upper airways (nose and throat) and thus these sites need to be explored to highlight the relationship between the different microbial ecosystems inhabiting the airways. To date, studies focusing on this question were mostly pathogen centered. They showed that PRPs from the environment are residents in the nasal cavity suggesting that this niche serves as a reservoir for further colonization via micro-aspiration in the lower airways [16,17]. PRPs persist in the nasal cavity and can re-colonize after antibiotic treatment giving raise to chronic infection in young CF patients [18,19]. However, to date, microbiome composition in the early stages of CF lung disease and the relationship between the microbiome at different niches of the upper and lower airways have not been studied in detail using next generation sequencing.

The objective of this study was to compare the microbiome of the nasal cavity, throat and sputum in young CF patients with mild to moderate lung disease. The first objective was to compare the three microbial ecosystems and determine potential differences in bacterial composition. The second objective was to explore the inter-individual variation between microbiomes in the upper and lower airways. We also compared the microbiome from young and adult CF patients to determine the onset and dynamics of abnormal microbiome composition in CF.

## Materials and Methods

### Pediatric CF patients

This prospective observational study was approved by the Ethics Committee of the University of Heidelberg and informed written consent was obtained from the patients, parents or legal guardians of all subjects. Airway samples from pediatric CF patients were obtained during routine visits every three months at periods of clinically stable lung disease or during acute pulmonary exacerbations. Patient characteristics are summarized in Table 1 and in S1 Table. In total, 98 samples (sputum, n = 32; nasal swabs, n = 36; throat swabs, n = 30) were analyzed. In all patients, the diagnosis of CF was based on characteristic clinical symptoms and confirmed by increased sweat Cl^-^ concentrations (≥60 mmol/L) and CFTR mutation analysis (S2 Table), and in 3 pancreatic-sufficient patients with borderline sweat test results by assessment of CFTR function in rectal biopsies according to established diagnostic criteria as previously described [20,21]. Anthropometric data of CF patients are provided in Table 1. Z-scores for weight, height, and BMI were derived from reference values of healthy children in Germany [22,23]. The status regarding infection with *Pseudomonas aeruginosa* was classified according to the following definition as negative, intermittent or chronic infection [24,25]: Patients were categorized as *P*. *aeruginosa* negative, when there was no growth of *P*. *aeruginosa* in the previous twelve month and titers of precipitating antibodies (alkaline protease, elastase and exotoxin A) (Mediagnost, Reutlingen, Germany) against *P*. *aeruginosa* antigens were negative. Intermittent *P*. *aeruginosa* infection was defined as culturing of *P*. *aeruginosa* in less than 50% of the samples in the last twelve month and negative antibodies. Chronic *P*. *aeruginosa* infection was defined as persistent presence of *P*. *aeruginosa* for at least 6 consecutive months, or less when combined with a positive finding (titer >1500) of two or more *P*. *aeruginosa* antibodies.

**Table 1:** Patients’ characteristics.

	mean ± SD (range) or n (%)
Number of patients	20
Age, years	16.1 ± 3.8 (7.0–22.0)
Male/female	8/12
F508del / F508del, n (%)	9 (45)
Pancreatic insufficiency, n (%)	17 (85)
Weight, kg	47.9 ± 9.4 (23.7–63.3)
Weight, SDS	-1.4 ± 1.3 (-4.1–0.1)
Height, cm	159.8 ± 11.9 (126.6–180.0)
Height, SDS	-1.3 ± 1.3 (-3.9–0.5)
BMI, kg/m^2^	18.6 ± 2.1 (14.8–23.2)
BMI, SDS	-0.8 ± 0.9 (-2.2–0.7)
FEV1, % pred.	70.5% ± 24.5% (33.9%–111.2%)
LCI	14.4 ± 2.9 (9.1–19.8)

BMI, body mass index; FEV1% pred, forced expiratory volume in 1 second % predicted; LCI, lung clearance index, SDS standard deviation score

### Adult CF patients cohort

Details of the adult CF patient cohort in this study were included in two previously published studies in which the airway microbiome was analyzed by extended aerobic and anaerobic bacterial culture, terminal restriction fragment length polymorphism (T-RFLP) analysis and high-throughput 454-FLX Titanium pyrosequencing of bacterial 16S rRNA marker genes present in sputum [26,27].

### Routine culture

All samples (ESwabs) were streaked and incubated for 24h and 48h at 36°C (6% CO_2_) on various media. Five media were used for aerobic isolation: Columbia-Agar (with 5% sheep blood) (BD Diagnostic, Heidelberg, Germany), Chocolate-Agar (BioMérieux, Nürtingen, Germany), McConkey- Agar (BioMérieux), Burkholderia cepacia-Spezial-Agar (7 days, 36°C) (BD), Sabouraud- Agar (7 days, 36°C) (BD). Additionally, two media were used for anaerobic isolation (36°C): Schaedler-Agar (BioMérieux) and Kanamycin-Vancomycin-Agar (BD). Anaerobic cultures were processed within an hour after receiving the sample. Once colonies were isolated, they were identified with a Microflex MALDI-TOF mass spectrometer (Bruker Daltonik, Bremen, Germany) [28].

### Sample pre-treatment

Samples were aliquoted (200μL) and treated with PMA^TM^ dye (Biotium Inc., Hayward, USA). PMA treatment modifies extracellular DNA from dead cells and avoids subsequent PCR amplification. 50μM of PMA dye was added to the aliquot and incubated for 5 min in the dark. The samples were then exposed to light (650 Watt, 20 cm distance to the samples) on ice and shaking by 100 rpm for 5 min to cross-link PMA to DNA. Viable cells were pelleted by centrifugation at 5000 x g for 10 min. Supernatant was removed and cells were recovered in 200 μL of sterile PBS and stored at -20°C until DNA extraction.

### DNA extraction

DNA extractions were performed using the QIAamp Mini Kit (QIAGEN, Hilden, Germany). Protease solution (7.2 mAU) and 200 μL of Buffer AL were added to the sample followed by a 15 sec vortex. Samples were incubated at 56°C for 10 min and then purified according to the manufacturer’s protocol. DNA was eluted by adding 100 μL of buffer AE to the column, incubation for 1 min at room temperature and centrifugation at 6000 x g for 1 min. Negative controls were performed by doing the extraction without clinical samples.

### Quantitative PCR

The number of 16S copies was quantified by quantitative PCR (qPCR) using Unibac primer (forward: 5′-TGG AGC ATG TGG TTT AAT TCG A-3′; reverse: 5′-TGC GGG ACT TAA CCC AAC A-3′). PCR reactions were performed in 15 μL volumes composed of 1X Sybr-green mastermix (Life technology, Darmstadt, Germany), 50 pmol of each primer and 2 μL of DNA (or plasmid DNA standards). The thermal cycler conditions were: a first denaturation at 95°C for 20 sec, 40 amplification cycles (95°C for 3 sec, 60°C for 30 sec) and two final steps at 95°C for 15 sec and 60°C for 1 minute followed by a melt curve. All reactions were performed in duplicate in a 7900HT Fast Real-time PCR system (Applied Biosystems, Foster City, USA). Quantification of the 16S number of copies was performed by comparison to the Cycle threshold value of a plasmid DNA standard which had been cloned in-house and quantified by spectrophotometry [29].

### Library preparation for next generation sequencing (NGS)

DNA was amplified using universal bacterial primers flanking the V6 region (v6L and v6R, taken from [30]). Each primer was tagged with an individual barcode to assign the sequences to the samples. PCR reactions were performed in 25 μL volumes composed of 1X FastStart Master mix (Roche Diagnostics Deutschland GmbH, Mannheim, Germany), 50 pmol of each primer and 2 μL of DNA. The thermal cycler (Primus 25, Peqlab Biotechnologie GmbH, Germany or FlexCycler², Analytik Jena AG, Germany) conditions were: a first denaturation at 94°C for 5 min, 32 amplification cycles (94°C for 45 sec, 52°C for 1 min and 72°C for 1 min 30 sec) and a final extension at 72°C for 10 minutes. PCR products were then checked on an agarose gel (2%) and concentrations were determined by comparing the intensity of the bands to intensity of the standards. Amplicons were purified by using a QIAquick PCR purification kit (QIAGEN). Purified products were checked for quality and concentration using a ND-1000 Nanodrop instrument (Nanodrop, Wilmington, USA) and Bioanalyser (Agilent Technologies Inc., Böblingen, Germany). Equimolar mix of all the PCR products (n = 98) was then sent to the Center for “Quantitative Analysis of Molecular and Cellular Biosystems” at Heidelberg University (BIOquant). Ligation of the sequencing adapters to the library was done by standard procedures and the library was paired-end sequenced on an Illumina Hiseq system.

A negative control for the extraction was done using sterile water instead of clinical sample to ensure that no contamination occurred. This negative control was then used as template during the PCR to verify absence of bacterial DNA. Furthermore, we also used sterile water as template for a negative control of the PCR reaction itself. No bands were observed when the negative controls were checked on an agarose gel.

### Analysis of sequences

Raw sequences were first filtered for quality; sequences with a quality score lower than 25 over 90 bp were discarded. The right and left paired-end sequences were then synchronized and orphan reads were discarded. The paired reads were merged in contigs and contigs were assigned to the sample with the barcodes on the right and left ends (allowing a mismatch of 1 nt per barcode). The assignment and the pre-treatment of the sequences were done using MOTHUR software (version 1.33) [31]. Sequences were screened for ambiguity in the sequences (maximum ambiguity allowed: 0) and for homopolymers (maximum homopolymer length allowed: 4 nt). Chimera detection was done by using the algorithm Uchime [32] which allows a fast, sensitive and accurate detection of chimera. Sequences were then clustered as Operational Taxonomic Units (OTU) (using the threshold of 3% of divergence) and OTU representative sequences were then classified at the taxonomic levels by comparisons with sequences from the SILVA database (cutoff set to 80% of similarity). Furthermore, OTU abundances for each sample were calculated to build an OTU table. All sequences data are available on MG-RAST server (MG-RAST IDs: XXXXX).

### Statistical analysis

An OTU table was used to calculate descriptive indices for alpha-diversity (non-parametric Shannon index), richness (Chao1 richness estimate) and evenness (Shannon index-based measure of evenness). Variation in the alpha-diversity among samples was tested with a linear mixed model with random effects for patients due to the inter-individual differences existing between patients. Paired comparisons were done with a post-hoc test [33,34]. Beta-diversity measures were performed to examine the differences between the samples. Principal component analysis (PCA) was performed directly on the OTU table to analyze how the samples were related. Distance matrices were constructed based on index of similarity of community membership and structure with distances and structure estimated with the Jaccard and Bray-Curtis indices, respectively. An analysis of similarities (ANOSIM) was performed to validate the statistical significance of the changes among samples. These changes were visualized by a PCoA (Principal coordinate analysis) and influences of each OTU on the differentiation of the samples were calculated by the Pearson correlation between the OTU abundance/presence and the coordinate of the samples on the different axis of the PCoA. We also performed a linear discriminant analysis (LDA) effect size (LEfSe) analysis to detect differentially abundant OTU between the samples. Correlation between OTU and clinical data was calculated with Pearson correlation between all variables and results were corrected for multiple testing. A mantel test between the similarity distance matrix (based on Bray-Curtis index and on Jaccard index) and a distance matrix based on culture data (euclidian distance) was performed to see if any relationship existed between microbiome and the detection of PRPs by culture. As inter-individual variation was important in our data, we assigned samples to ecotypes using a Dirichlet multinomial mixture (DMM) model [35] and analyzed the relationships between partitioning and the clinical data. The ecotype assignment clustered similar samples in the same partition. We considered in this model that microbial communities sharing a high degree of similarity belong to the same community type (ecotype). We calculated the accuracy of this clustering using the Dirichlet probabilistic distribution [35]. For each categorical clinical data, we tested the association on a contingency table using Fisher’s exact test and continuous data were tested using ANOVA. All statistical analyses were performed with MOTHUR 1.33.0 and R 3.0.2.

## Results

### Quality of the sequencing dataset from CF samples

**Fig 1:**
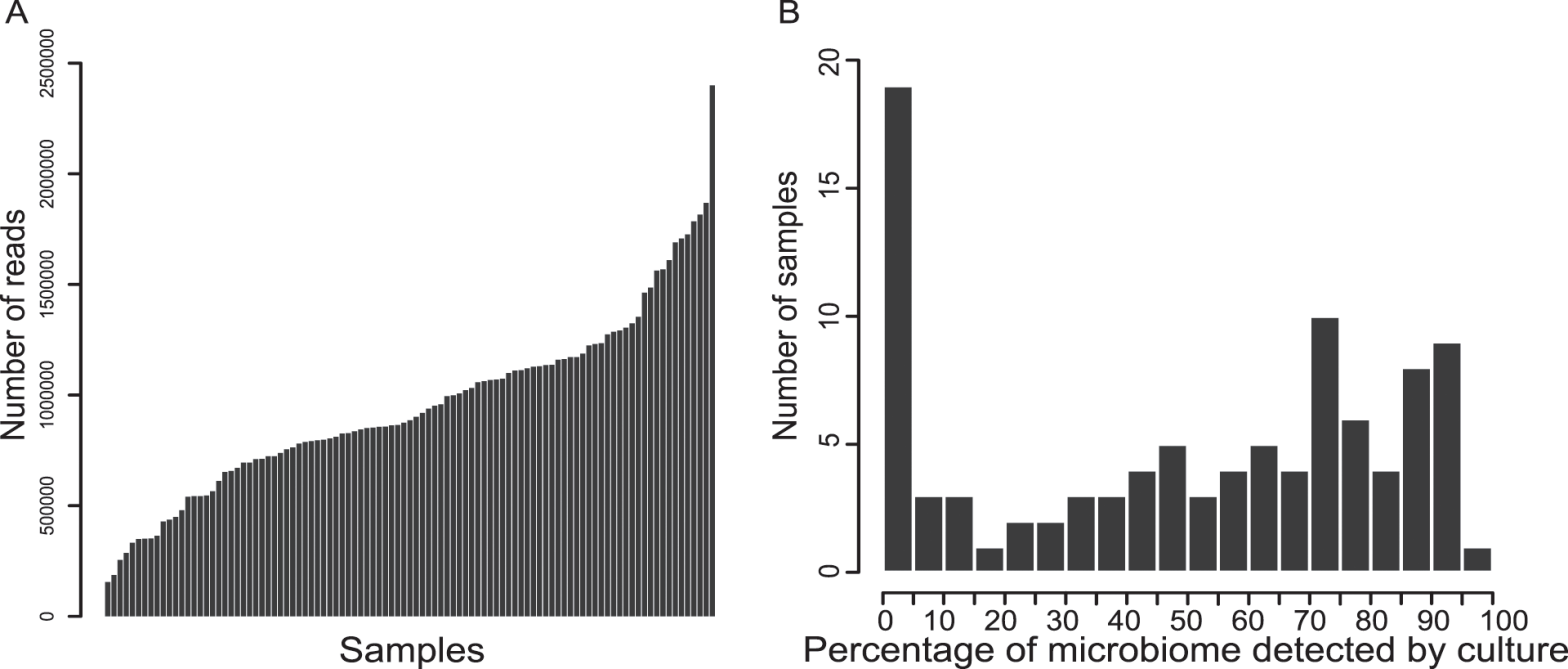
Descriptive analysis of the sequencing reads. (A) Distribution of the number of reads over the whole dataset. (B) Number of samples with detection of the indicated percentage of the microbiome at the genus level by culture. Strains isolated by culture were classified at the genus level and correspondence with the NGS dataset was analyzed.

**Fig 2:**
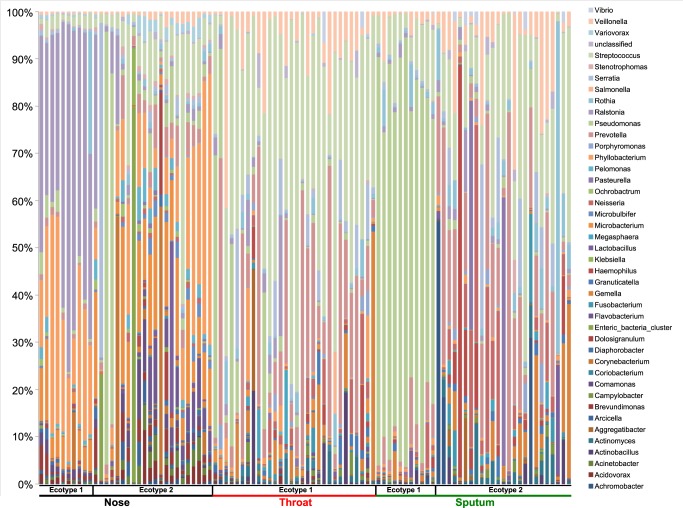
Relative abundance of the bacterial genera depending on the sampling site. The colored segments of each bar represent the proportion of reads mapping to different bacterial genera.

A total of 122,611,422 reads were obtained and passed through quality filter and chimera detection resulting in 94,177,638 (76.8%) clean and non-chimeric reads. These reads were unevenly distributed among the samples (Fig. 1A) and the whole data set was normalized by subsampling the same number of reads for each sample (154,849 reads per sample). Reads were clustered in 295,617 OTUs (using a threshold of 97% identity) but most of the OTUs were represented by few reads. We sorted the data to filter any OTUs that did not reach 0.001% of the sample microbiome. Thus, 76 OTUs were retained and assigned to the taxonomic level (Fig. 2, S1 Fig.). Taxonomy was validated or improved by a blast on NCBI database (using a threshold of 99% identity). The coverage of our sequencing was assessed by rarefaction curves and computing the Good’s estimator of coverage (S1 Table, S2 Fig.). Each sample’s curve reached a plateau and the Good’s estimator of coverage was always higher than 94%.

### Comparison of NGS with culture analysis

In total, 18 genera were found by culture as compared to 76 frequent OTUs by NGS. From all specimens (nose, throat and sputum) included in the study, only two samples coming from the nose were classified as sterile with no aerobic or anaerobic bacteria detected by culture. The other samples were positive for one to seven different genera. We compared the number of genera detected by culture with the relative abundance of those genera in the NGS dataset (Fig. 1B); of 98 samples, 45 showed less than 50% detection by culture as compared to NGS. These samples were mostly colonized by OTUs including *Phyllobacterium sp*., *Ralstonia sp*., *Microbacterium sp*. *or Corynebacterium sp*. that we failed to detect by culture despite the numerous media and conditions used. In contrast, in 38 samples, over 70% of genera detected by NGS were also detected by culture. These samples were colonized by classical PRPs such as *Staphylococcus sp*., *Pseudomonas sp*., and anaerobic genera such as *Prevotella sp*. and *Veillonella sp*. that our aerobic and anaerobic culture methods can easily detect. Overall, the mantel test comparing microbiome data obtained by NGS and PRPs detection by culture showed a significant but weak correlation between the two datasets (*r* = 0.30, *p-value* < 0.001).

### Microbiome analysis in clinically stable children with CF

**Fig 3:**
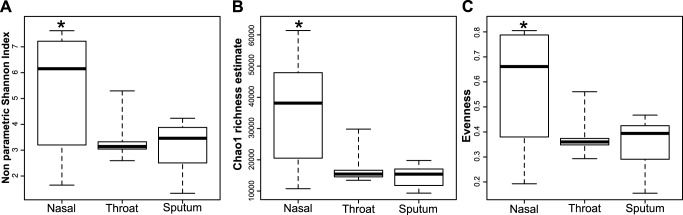
Alpha-diversity of the upper and lower airways microbiomes from clinically stable children with CF. Alpha-diversity was calculated with the non parametric Shannon index (A), richness was estimated with the Chao1 estimate (B) and evenness was calculated based on the Shannon index (C). Alpha-diversity variation among nose, throat and sputum microbiome was analyzed with a linear mixed model with random effects for CF patients and paired comparisons were done with a Tukey post-hoc test for pairwise comparison.

We first analyzed samples that were obtained from pediatric CF patients with clinically stable lung disease obtained during routine visits. The alpha-diversity differed among the samples and between the three sampling sites (Fig. 3A) with the nasal microbiome showing a higher diversity compared to throat and sputum microbiomes. The richness and the evenness were analyzed to see if the observed divergence was consistent on these two components of the alpha-diversity (Fig. 3B, C). The pattern observed was the same for the three analyses with a divergence of the nasal microbiome (p<0.01) and no difference between throat swabs and sputum. These analyses demonstrated that microbial communities from the nose were characterized by an even polymicrobial assemblage, while sputum and throat microbiomes showed lower evenness and diversity. In nasal samples, a negative correlation (*r* < -0.4, *p-value* <0.05) was found between evenness and three different OTUs classified as *Enterobacteriaceae*, *Ralstonia sp*. and *Pseudomonas aeruginosa*. In contrast, a positive correlation (*r* > 0.4, *p-value* < 0.05) was found between evenness and seven OTUs belonging to *Variovorax sp*., *Acinetobacter sp*., *Flavobacterium sp*. (2 OTUs), *Microbulbifer sp*., *Acidovorax sp*. and *Pelomonas sp*.

### Increased bacterial load in CF patients is correlated with decreased diversity

**Fig 4:**
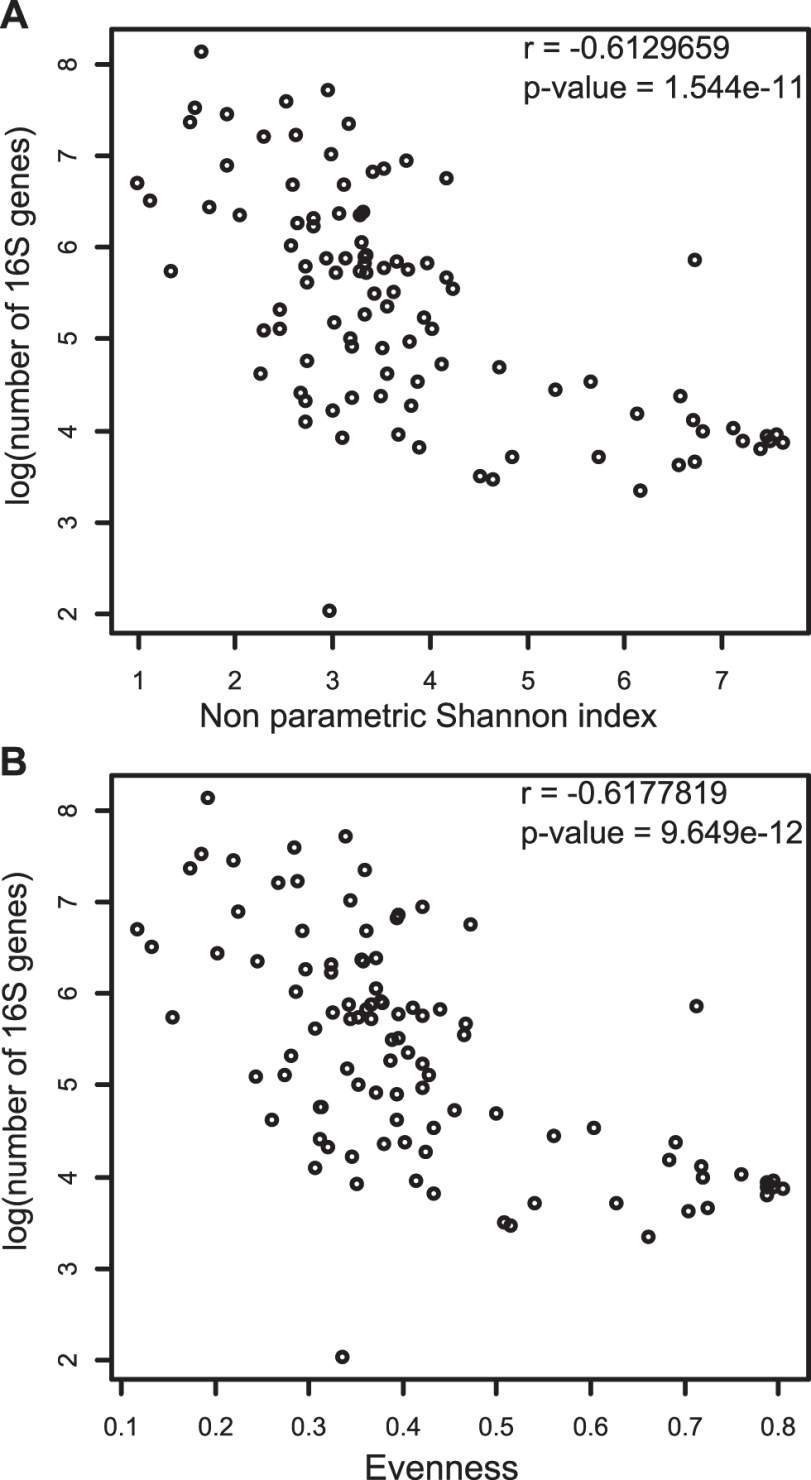
Correlation between alpha-diversity and bacterial load in CF airways. The alpha-diversity is represented by the non parametric Shannon index (A) and the evenness index based on the Shannon index (B). The microbial load was measured via the proxy of the number of 16S genes. Samples from the three sampling sites are represented.

A negative correlation was found between the total bacterial load, as assessed by numbers of copies of 16S gene, and both the non-parametric Shannon index (*r* = -0.61, *p-value* = 1.544e-11) (Fig. 4A) and evenness (*r* = -0.62, *p-value* = 9.649e-12) (Fig. 4B) indicating that increased microbial colonization is associated with a decrease in diversity. This finding is supported by the fact that evenness decreased with acute pulmonary exacerbations when a PRP (*P*. *aeruginosa* in our study) was detected. No correlation was found between the number of copies of 16S and the chao1 richness estimate (*r* = 0.06, *p-value* = 0.6928). Despite results found in adults [12], we found no correlation between richness (chao1 richness estimate: *r* = 0.04, *p-value* = 0.7805), evenness (*r* = -0.13, *p-value* = 0.3934) or overall alpha-diversity (*r* = -0.13, *p-value* = 0.4084) with patient age in our pediatric CF cohort (data not shown).

### Nasal CF microbiome structure differs from throat and sputum microbiome

**Fig 5:**
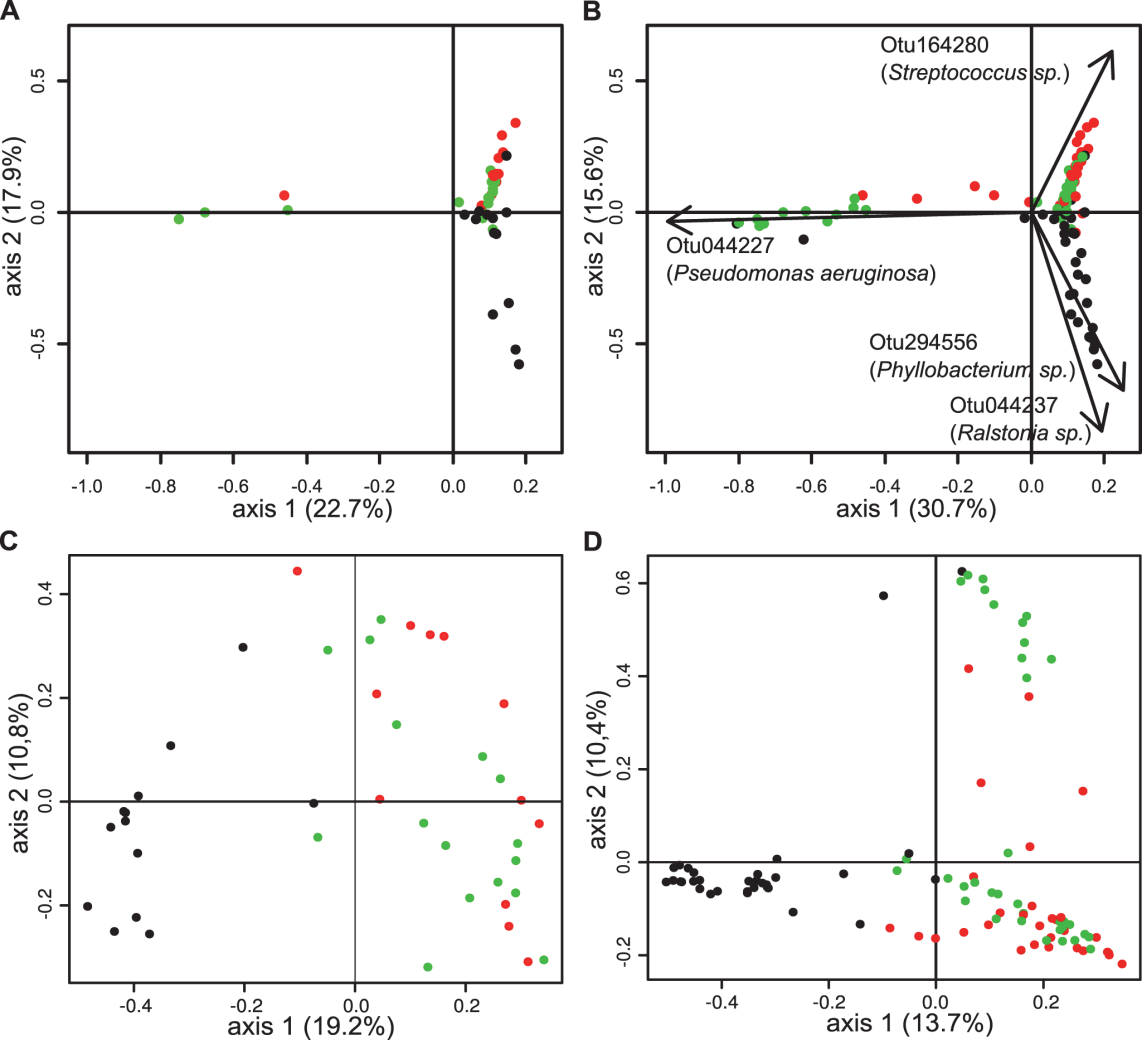
Spatial analysis of the airways microbiome. Nasal swabs are represented by black dots, throat swabs by red dots, sputum samples by green dots. (A) Principal Component Analysis of samples obtained from clinically stable children with CF. In panel (B) samples from patients during exacerbation were added. Pearson correlations were performed to highlight which OTUs were responsible for the divergence among the samples. Correlation was considered significant when the coefficient of correlation was higher than 0.6 and p-value < 0.01. (C) Principal Coordinates Analysis was performed on samples obtained from clinically stable CF patients or (D) on all available samples from CF patients irrespective of the clinical status.

As the three sampling sites showed divergence in the alpha-diversity, we next analyzed the beta-diversity to depict this divergence and assess its accuracy. The principal component analysis (PCA) showed that nasal samples from clinically stable CF patients can be easily discriminated from throat and sputum samples (Fig. 5A). Discrimination was even stronger if we included samples collected during exacerbation (Fig. 5B). Axis 1 in the PCA did not completely discriminate the three sampling sites but was the most discriminating axis among samples. This axis was correlated strongly with the abundance of one OTU classified as *P*. *aeruginosa*. Axis 2 was the most discriminating dimension regarding the three sampling sites and is correlated with three OTUs. Two OTUs, namely *Phyllobacterium sp*. and *Ralstonia sp*., were mostly abundant in nasal samples while the third OTU, *Streptococcus sp*., was more prevalent in throat and sputum samples. The same result was found with PCoA based on the Bray-Curtis index. Bray-Curtis index takes into account the abundance of each OTU (i.e. structure of the microbiome) and estimates of the dissimilarity of the samples. This analysis also showed a divergence between the structure of the microbiome in the nose and both throat and sputum (Fig. 5C and 5D). Furthermore, an analysis of similarities (ANOSIM) showed that nose microbiomes are significantly different from the two others (r-value = 0.663 and 0.63, *p-value* < 0.001), while microbiomes from throat and sputum did not differ (r-value = 0.039, *p-value* = 0.237).

A PCoA based on the Jaccard index was performed to analyze differences in the composition of the different microbiomes. The Jaccard index is a similarity coefficient which does not take into account the abundance of the OTUs but rather relies on presence/absence of OTUs (i.e. the composition of the microbiome). No differences were observed on the PCoA based on Jaccard index. ANOSIM analysis confirmed this observation with no overall significant variation among the three sampling sites (R-value = 0.002, *p-value* = 0.423).

### OTU differences between nasal, throat and sputum microbiomes in CF patients

The divergence between nasal and throat microbiomes was based upon 28 OTUs from the 76 most abundant OTUs classified in 23 genera (Table 2). The top 5 abundant OTUs in the nasal microbiome were Otu044237 (*Ralstonia sp*., 17.5%), Otu093761 (*Microbacterium sp*., 14.1%), Otu294556 (*Phyllobacterium sp*., 13.4%), Otu164280 (*Streptococcus sp*., 10.1%), Otu042022 (Enteric Bacteria cluster, 6.3%). The throat microbiome was dominated by two major OTUs; Otu164280 (*Streptococcus sp*., 24.1%) and Otu263762 (*Prevotella sp*., 18%). The divergence between the nasal and sputum microbiomes was based upon 28 OTUs classified in 23 genera (Table 2). The sputum microbiome was dominated by two OTUs: Otu044227 (*P*. *aeruginosa*, 15.1%) and Otu263762 (*Prevotella sp*., 9.6%). The same divergent OTUs were found between the nose and both sputum and throat (21 divergent OTUs in common) strengthening the close relationship between throat and sputum. This result is confirmed by the small numbers of OTUs found divergent between throat and sputum with only eight OTUs present in low abundance except for the *Streptococcus* genus (Table 2).

**Table 2:** Differential abundance of the most abundant OTUs among the different sampling sites.

OTU	difference between nose and sputum	difference between nose and throat	difference between sputum and throat	Taxonomy
Otu009303	no	Throat	Throat	Firmicutes;Bacilli;Lactobacillales;Streptococcaceae;Streptococcus
Otu032767	Sputum	Throat	No	Firmicutes;Bacilli;Lactobacillales;Carnobacteriaceae;Granulicatella
Otu039334	no	Nose	No	Actinobacteria;Actinomycetales;Corynebacteriaceae;Corynebacterium
Otu040700	Sputum	Throat	No	Actinobacteria;Actinobacteria;Coriobacteriales;Coriobacteriaceae;Atopobium
Otu042022	no	Nose	No	Proteobacteria;Gammaproteobacteria;Enterobacteriales;Enterobacteriaceae;Enteric_Bacteria_cluster
Otu044227	no	no	No	Proteobacteria;Gammaproteobacteria;Pseudomonadales;Pseudomonadaceae;Pseudomonas;aeruginosa
Otu044237	Nose	Nose	No	Proteobacteria;Betaproteobacteria;Burkholderiales;Burkholderiaceae;Ralstonia
Otu048236	Sputum	no	No	Actinobacteria;Actinomycetales;Micrococcineae;Micrococcaceae;Rothia
Otu050175	Nose	Nose	Throat	Proteobacteria;Betaproteobacteria;Burkholderiales;Comamonadaceae;Pelomonas
Otu054786	Nose	Nose	No	Proteobacteria;Alphaproteobacteria;Caulobacterales;Caulobacteraceae;Brevundimonas
Otu059814	Sputum	no	Sputum	Actinobacteria;Actinomycetales;Micrococcineae;Micrococcaceae;Rothia
Otu063017	Nose	Nose	Throat	Proteobacteria;Betaproteobacteria;Burkholderiales;Comamonadaceae;Variovorax
Otu093761	Nose	Nose	No	Actinobacteria;Actinomycetales;Micrococcineae;Microbacteriaceae;Microbacterium
Otu106057	Nose	Nose	No	Proteobacteria;Gammaproteobacteria;Pseudomonadales;Moraxellaceae;Acinetobacter
Otu110859	Nose	Nose	No	Firmicutes;Bacilli;Lactobacillales;Lactobacillaceae;Lactobacillus;sakei
Otu113401	Sputum	Throat	No	Firmicutes;Clostridia;Clostridiales;Veillonellaceae;Veillonella
Otu133067	Nose	Nose	no	Proteobacteria;Betaproteobacteria;Burkholderiales;Comamonadaceae;Comamonas
Otu134395	Nose	Nose	no	Bacteroidetes;Flavobacteria;Flavobacteriales;Flavobacteriaceae;Flavobacterium
Otu164280	no	Throat	Throat	Firmicutes;Bacilli;Lactobacillales;Streptococcaceae;Streptococcus
Otu172150	Nose	Nose	no	Proteobacteria;Betaproteobacteria;Burkholderiales;Comamonadaceae;Diaphorobacter
Otu211292	Nose	Nose	no	Proteobacteria;Gammaproteobacteria;Alteromonadales;Alteromonadaceae;Microbulbifer
Otu220996	Nose	no	Throat	Proteobacteria;Betaproteobacteria;Burkholderiales;Comamonadaceae;Acidovorax
Otu222581	Sputum	Throat	no	Firmicutes;Clostridia;Clostridiales;Veillonellaceae;Veillonella
Otu241870	Nose	Nose	no	Proteobacteria;Gammaproteobacteria;Xanthomonadales;Xanthomonadaceae;Stenotrophomonas
Otu242798	Nose	Nose	no	Proteobacteria;Alphaproteobacteria;Rhizobiales;Brucellaceae;Ochrobactrum
Otu252790	Sputum	no	no	Fusobacteria;Fusobacteria;Fusobacteriales;Fusobacteriaceae;Fusobacterium
Otu259630	Nose	Nose	no	Bacteroidetes;Sphingobacteria;Sphingobacteriales;Cytophagaceae;Arcicella
Otu260625	Nose	Nose	Throat	Proteobacteria;Betaproteobacteria;Burkholderiales;Comamonadaceae;Pelomonas
Otu261770	no	no	Throat	Firmicutes;Bacilli;Lactobacillales;Streptococcaceae;Streptococcus
Otu263762	no	no	no	Bacteroidetes;Bacteroidia;Bacteroidales;Prevotellaceae;Prevotella
Otu264082	Sputum	Throat	no	Proteobacteria;Epsilonproteobacteria;Campylobacterales;Campylobacteraceae;Campylobacter
Otu265054	Nose	Nose	no	Proteobacteria;Betaproteobacteria;Burkholderiales;Comamonadaceae;Comamonas
Otu275373	Nose	Nose	no	Bacteroidetes;Flavobacteria;Flavobacteriales;Flavobacteriaceae;Flavobacterium
Otu276093	Sputum	Throat	no	Firmicutes;Bacilli;Bacillales;Family_XI_Incertae_Sedis;Gemella
Otu294556	Nose	Nose	no	Proteobacteria;Alphaproteobacteria;Rhizobiales;Phyllobacteriaceae;Phyllobacterium

Significance of the difference was calculated with LEfSe algorithm (FDR correction) and the site with the higher abundance is indicated. OTUs showing no differences between all the sites are not represented in the table except for two most abundant OTUs (Otu044227 and Otu263762).

### Comparison of microbiome composition in pediatric versus adult CF patients

**Fig 6:**
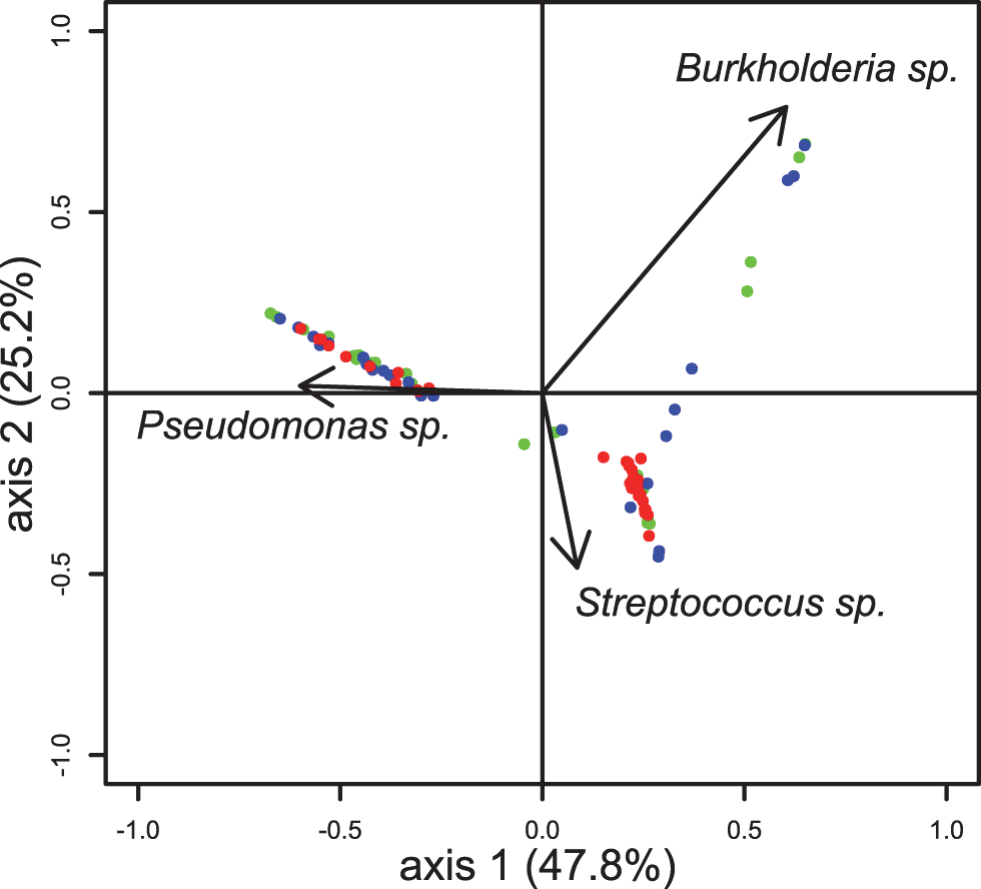
Principal Component Analysis of sputum samples from children and adult patients CF patients. Sputum samples from children with CF are represented by red points and adult CF patients by blue (at the time of admission in the clinic) and green points (after antibiotics treatment). Pearson correlations were performed to highlight which genera were responsible for the divergence among the samples. Correlation was considered significant when the coefficient of correlation was higher than 0.6 and p-value < 0.01.

In order to explore if this inter-individual variation observed between CF patients was age-dependent, we compared our data obtained from sputum samples from pediatric CF patients with data from a cohort of adult CF patients [26,27]. Each OTU was classified at the genus level and both datasets were compared by means of a PCA. This comparison showed striking convergence, except for one group of adult patients highly infected by *Burkholderia*. Similar to pediatric CF patients, the adult CF cohort showed a clustering in different ecotype dominated either by *Pseudomonas* or *Streptococcus*. Furthermore, high inter-individual variation was also found in adults with patients dominated by either *Pseudomonas*, *Streptococcus* or *Burkholderia* (Fig. 6).

### Ecotype assignment identifies chronic Pseudomonas infection as decisive variable for microbiome diversity

**Fig 7:**
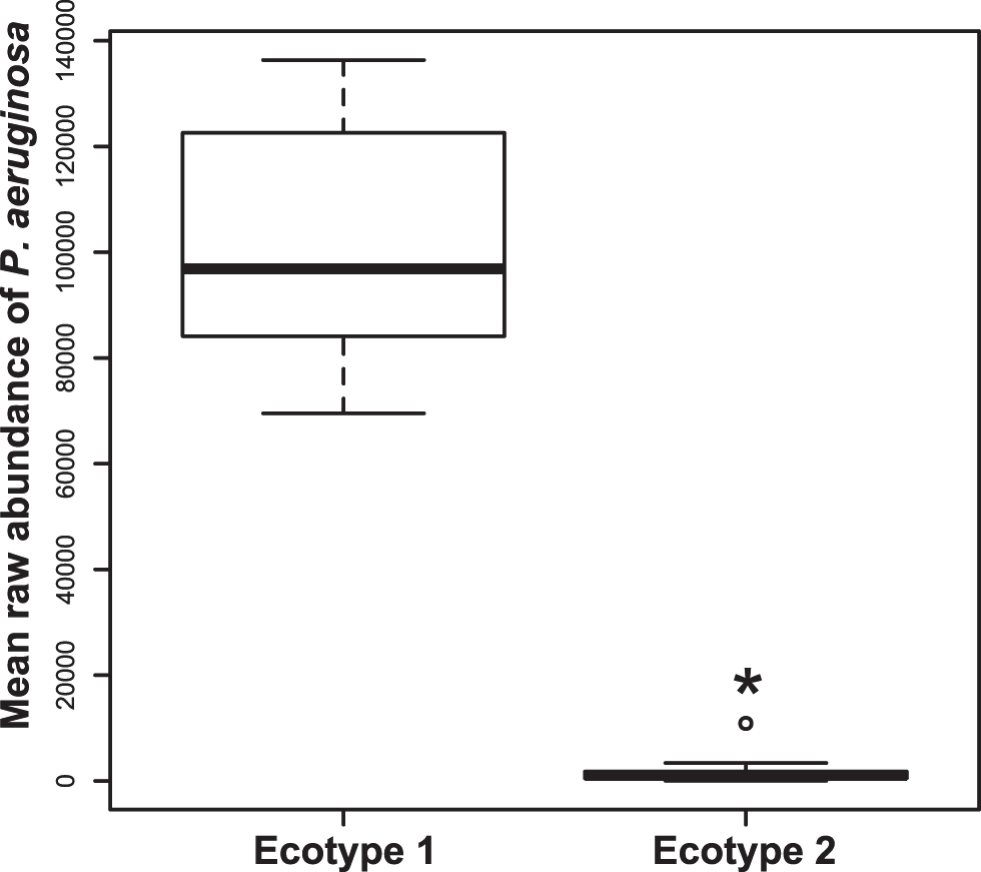
Differential mean raw abundance of Pseudomonas aeruginosa between the two ecotypes of sputum samples. Mean raw abundance of *Pseudomonas aeruginosa* in sputum samples from both ecotypes are represented by the raw numbers of reads in the sample. Statistical differences were assessed by a Wilcoxon test.

The ecotype assignment was consistent with the PCA results. Two partitions were found for both samples coming from nose and sputum reflecting the inter-individual variability observed in the PCA and only one cluster for the throat samples. The nasal partitions were different mostly regarding the diversity and the evenness. Some patients (ecotype 2) exhibited a highly diverse and even nasal microbiome while other patients (ecotype 1) showed low diversity microbiome dominated by two OTUs; Otu044237 (*Ralstonia sp*.) and Otu294556 (*Phyllobacterium sp*.) which accounted for 74.3% of the microbiome. The same pattern was observed in the sputum partitioning, with some patients (ecotype 2) possessing a highly diverse and even microbiome where the predominant OTUs belonged to *Streptococcus sp*. and *Prevotella sp*. and did not exceed 10% of the microbiome. In contrast, patients in ecotype 1 were primarily infected by one OTU (Otu044227; *P*. *aeruginosa*) which accounted for 73.4% of the microbiome (Fig. 7). We found a strong correlation (*p-value* < 0.001) between the sputum ecotype assignment and the classification of chronic infection by *P*. *aeruginosa*. Of note, patients belonging to sputum ecotype 1 were most likely classified as patients suffering of chronic infection with *Pseudomonas aeruginosa*.

## Discussion

Human airways are characterized by interconnections between highly compartmentalized environmental niches (nose, throat and lungs). In this study, we used, for the first time to our knowledge, massive parallel sequencing to determine relationships between different compartments of the upper and lower airways, i.e. nose, throat and sputum, in children and adolescents with CF. A general observation was the weak concordance between culture-dependent and NGS analysis of the microbiome (Fig. 1). Limitations in handling and processing of the samples may account for the differences as some studies showed that a high percentage of the sputum microbiome can be cultured under optimized conditions [27,36,37]. However, our results suggest that samples analyzed within a routine setting at a university microbiology laboratory lack a considerable number of bacteria and that NGS analysis may be superior for diagnostic characterization of polymicrobial CF airways infections in the near future.

Our first important result is that the microbiome composition in the nose differed considerably from corresponding throat and sputum samples. This divergence was based on both the alpha-diversity and the structure of the microbial community (Fig. 3 and Fig. 5). This divergence between upper airways and lower airways in CF patient is concordant with the findings of previous studies using culture-dependent analysis in CF or NGS in healthy patients [11,38]. As the nasal cavity is the first compartment of the respiratory system that is in close contact with the environment this might account for higher diversity. This assumption of an environmental origin for bacterial colonization was previously detailed in other studies focusing on *P*. *aeruginosa* [16,19] which support our hypothesis. Previous reports showed that the nasal cavity and paranasal sinuses can serve as a reservoir for PRPs, thus building a niche for PRP’s in a highly polymicrobial community with few selection pressures from antibiotic treatment [16,39]. Nose microbiota is highly even regarding the membership (median evenness = 0.66) indicating that competition among the different bacteria is low or reaches a stable state as found in healthy children [40]. The same pattern was observed in our CF cohort with a significant reduction in evenness during exacerbation (0.66 to 0.41) indicating that evenness in the nasal cavity is important for stability. When evenness was decreased, nasal cavities were over-colonized by three different OTUs depending on the patient (Enteric bacteria, *Ralstonia sp*. and *P*. *aeruginosa)*. This indicates that when dysbiosis occurs opportunistic bacteria overgrow. Interestingly, the three major competitors observed in our study are known to be pathogens in CF patients [19,39,41–43]. Of note, the most abundant OTUs found in the nose belong to known typical or atypical CF pathogens, the latter comprising *Ralstonia sp*., *Phyllobacterium sp*. and *Microbacterium sp*. As those bacteria are environmental bacteria and possible contaminants, we examined correlation between those bacteria and biomass. If those bacteria were only present in low biomass samples, this might indicate contamination. However, no correlations were found between biomass and the abundance of those bacteria and a variety of internal negative controls were indeed negative. These results suggest that CF pathogens are already contained in the upper airways microbiome at times when patients are clinically stable and may expand when local defense mechanisms are impaired and then contribute to lower airways infection. This result is strengthened by a previous study of Maughan *et al*. who found those bacteria in surgically resected lungs from CF and non-CF patients [44].

We also demonstrate that the microbiome composition of the throat and the sputum were overall highly similar (Fig. 5). However, we found eight OTUs which are discriminant between throat and sputum. The high similarity between throat and sputum raises the question about contamination of sputum from the throat. Previous studies have demonstrated that CF sputum and oral samples had distinct bacterial communities supporting the hypothesis that oral contamination has a minimal impact on the assessment of airway samples from CF patients [45]. Furthermore, precautions were taken to control for contamination in two important steps of the molecular process, namely extraction of the DNA and PCR reaction.

Indeed in stable patients characterized by the sputum ecotype 2, throat and sputum microbiome are closely related and present a more diverse and stable community less prone to chronic infection by *P*. *aeruginosa*. This microbiota is dominated by *Streptococcus*, *Prevotella* and *Veillonella*. Those genera were also characterized as classical commensal of the lung in healthy non-smokers in a previous study [10]. For those patients our results suggest that throat sampling may be a good surrogate in clinically stable patients without chronic *P*. *aeruginosa* infection. Patients belonging to the sputum ecotype 1 on the other side showed a decreased similarity between throat and sputum. This decrease is due to the high disturbance in the sputum while the throat microbiota seems to be more resistant. In the sputum, we observed an increase of *P*. *aeruginosa* and a decrease in the alpha-diversity. This interpretation is also supported in our study by the negative correlation between the number of 16S copies found in the sample and the alpha-diversity indicating that the over-growth of one species greatly increases bacterial load and disturbs the stability of the microbiota (Fig. 2). This scenario has also been observed for other CF pathogens (*Achromobacter xylosoxidans*, *Pandoreae apista*), where the alpha-diversity decreased due to the over-growth of one species, which in turn was associated with poorer lung function [46,47].

In adults, some patients showed a microbiota highly similar to the ecotype 1 observed in children while other were subject to an over-growth of *P*. *aeruginosa* or *Burkholderia sp*. in a subgroup of infected patients resulting in a third ecotype. Previous reports already showed that pathogens from the *Burkholderia cepacia* complex are more prevalent in adults than children [19]. The fact that children with CF exhibit a microbiome close to adult CF patients indicates that the abnormal microbiome observed in adult CF patients compared to healthy people is probably established early in life. However, future longitudinal studies will be required to determine the sensitivity and specificity of microbiome analyses in distinguishing intermittent from chronic *Pseudomonas* infection in patients with CF (Fig. 6).

In conclusion, we demonstrate that the microbiome of the nose differs substantially from the throat and the lower airways in children with CF. Further, we demonstrate that the microbiomes of the throat and sputum are closely related in clinically stable children with CF indicating that throat swabs may be a surrogate for the lower airways microbiome in *Pseudomonas* negative young children with CF who are not able to expectorate sputum. Finally, we demonstrate that an abnormal microbiome characteristic of adult CF patients with advanced lung disease is already established in children with CF, suggesting that changes in microbiome structure may occur early in the pathogenesis of CF lung disease. Analysis of the sputum microbiome may be informative for discrimination between intermittent and chronic infection with *Pseudomonas aeruginosa*, an issue that has important diagnostic and therapeutic implications but remains difficult to solve by culture-dependent diagnostics [24,48,49]. In future work, it will therefore be interesting to study longitudinal samples from the different airway niches in patients with CF to determine whether PRPs established in the upper airways can subsequently colonize the lungs and whether this depends on the microbiome ecotype.

## Supporting Information

S1 Fig Relative abundance of the bacterial genera depending on the sampling site. Proportions of bacterial taxa in each sample type. Each column corresponds to an individual respiratory tract. The type of samples is indicated at the bottom of each cluster. Each row represent a specific OTU identified by it taxonomic assignment. OTUs with the same taxonomic assignment result in duplicate rows. Rows were submitted to a hierarchical clustering to highlight the taxa that show similar patterns. Columns were clustered depending on the sample type. The relative abundance of each taxa is represented by the color code (key to the left). The absolute number of 16S rRNA copies evaluated by qPCR is shown along the bottom.
(TIF)

S2 Fig Rarefaction curves of the different sampling sites. Rarefaction curves were constructed with the dataset containing the abundant OTUs (>0.001% of the microbiome).
(TIF)

S1 Table Descriptive statistics of the samples. (DOCX)

S2 Table Summary of CFTR genotypes with pancreatic status of patients with cystic fibrosis. (DOCX)
