# Supplementary figures and images for: Comparison of Microbiomes from Different Niches of Upper and Lower Airways in Children and Adolescents with Cystic Fibrosis
Source: PLoS One. 2015 Jan 28;10(1):e0116029. doi: 10.1371/journal.pone.0116029 (PMC4309611; doi:10.1371/journal.pone.0116029)

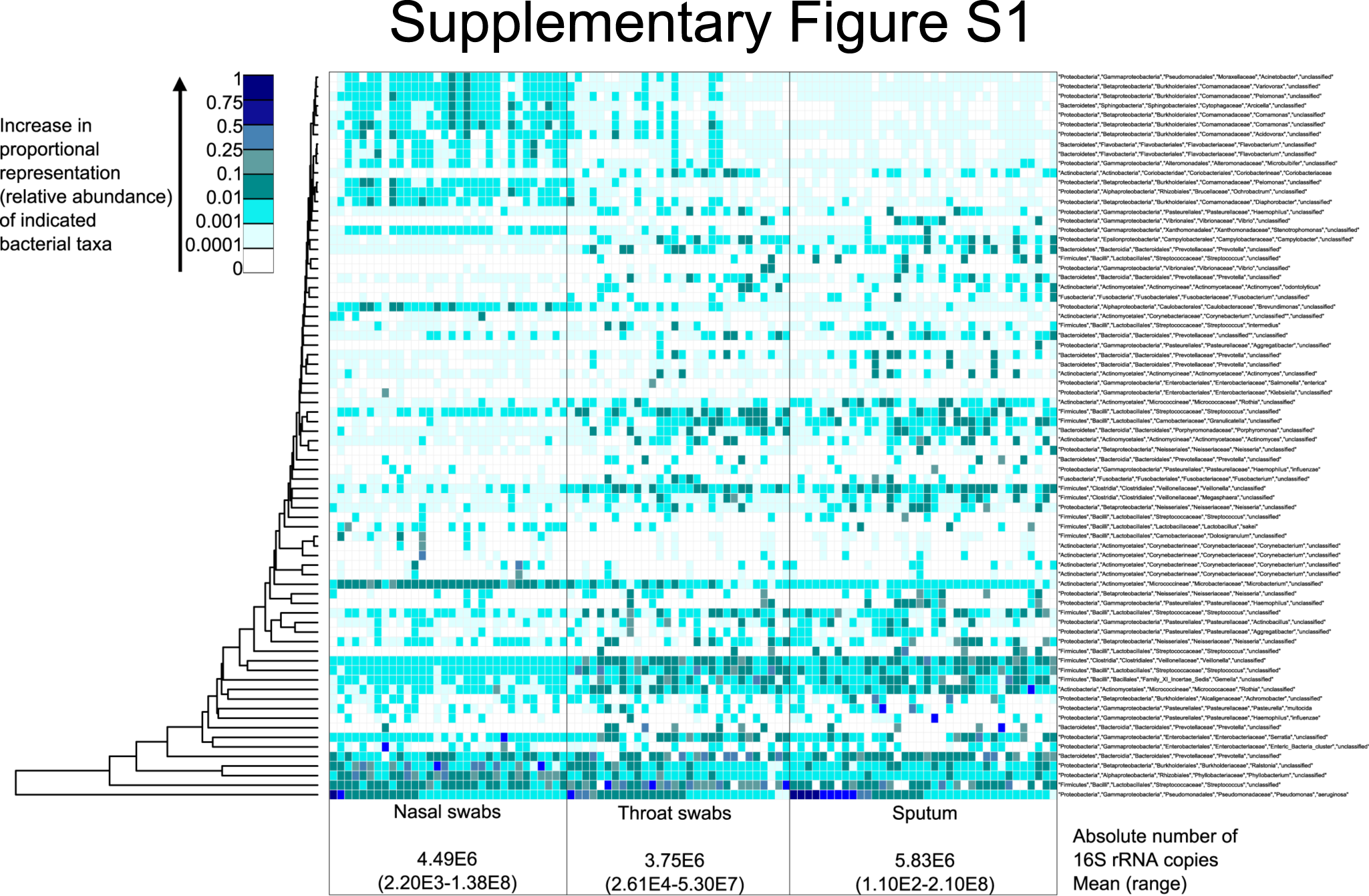

Supplement: S1 Fig — Proportions of bacterial taxa in each sample type. Each column corresponds to an individual respiratory tract. The type of samples is indicated at the bottom of each cluster. Each row represent a specific OTU identified by it taxonomic assignment. OTUs with the same taxonomic assignment result in duplicate rows. Rows were submitted to a hierarchical clustering to highlight the taxa that show similar patterns. Columns were clustered depending on the sample type. The relative abundance of each taxa is represented by the color code (key to the left). The absolute number of 16S rRNA copies evaluated by qPCR is shown along the bottom. (TIF) [file pone.0116029.s001.tif]

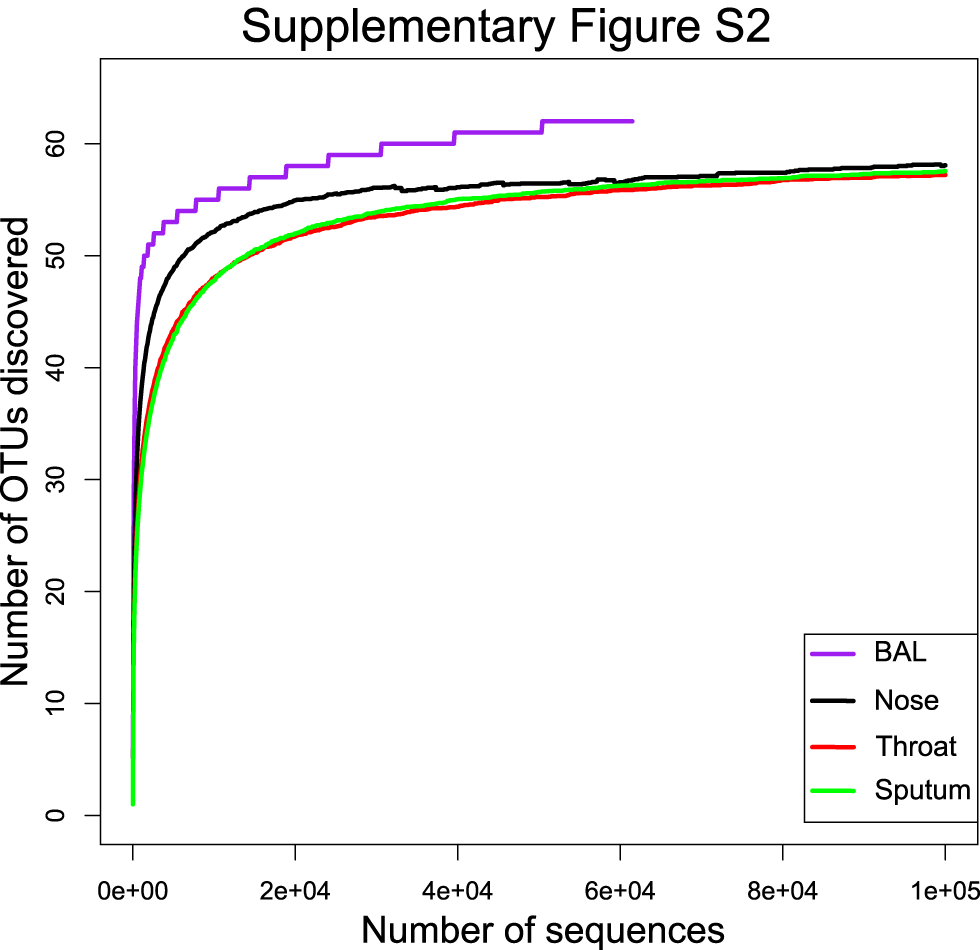

Supplement: S2 Fig — Rarefaction curves were constructed with the dataset containing the abundant OTUs (>0.001% of the microbiome). (TIF) [file pone.0116029.s002.tif]
