# Supplementary material for: Comparison of Microbiomes from Different Niches of Upper and Lower Airways in Children and Adolescents with Cystic Fibrosis
Source: PLoS One. 2015 Jan 28;10(1):e0116029. doi: 10.1371/journal.pone.0116029 (PMC4309611; doi:10.1371/journal.pone.0116029)
Supplement: S1 Table — (DOCX) [file pone.0116029.s003.docx]

**Supplementary Table 1.** *Descriptive statistics of the samples.*

| Sample number | patient number | Good's estimator of coverage | Number of abundant OTUs (>0.001%) | age | gender | sample | status |
| --- | --- | --- | --- | --- | --- | --- | --- |
| 2 | 13905436 | 0.999985 | 55 | 18 | female | throat swab | routine |
| 3 | 13905436 | 0.99998 | 60 | 18 | female | nasal swab | routine |
| 4 | 13905436 | 0.999986 | 59 | 18 | female | Sputum | routine |
| 17 | 14967982 | 0.999969 | 57 | 15 | female | throat swab | exacerbation |
| 18 | 14967982 | 0.99997 | 58 | 15 | female | nasal swab | exacerbation |
| 19 | 14967982 | 0.999968 | 53 | 15 | female | Sputum | exacerbation |
| 30 | 18709406 | 0.999968 | 54 | 19 | female | throat swab | exacerbation |
| 31 | 18709406 | 0.999979 | 58 | 19 | female | nasal swab | exacerbation |
| 32 | 18709406 | 0.999992 | 60 | 19 | female | Sputum | exacerbation |
| 38 | 9765735 | 0.999993 | 56 | 16 | female | throat swab | exacerbation |
| 39 | 9765735 | 0.999978 | 58 | 16 | female | nasal swab | exacerbation |
| 40 | 9765735 | 0.999986 | 60 | 16 | female | Sputum | exacerbation |
| 41 | 14338705 | 0.999979 | 59 | 17 | male | throat swab | exacerbation |
| 42 | 14338705 | 0.999973 | 61 | 17 | male | nasal swab | exacerbation |
| 43 | 14338705 | 0.999971 | 58 | 17 | male | Sputum | exacerbation |
| 44 | 14967982 | 0.999978 | 57 | 15 | female | throat swab | end of antibiotic treatment |
| 45 | 14967982 | 0.999983 | 59 | 15 | female | nasal swab | end of antibiotic treatment |
| 46 | 14967982 | 0.999977 | 60 | 15 | female | Sputum | end of antibiotic treatment |
| 47 | 10653207 | 0.999992 | 60 | 15 | male | throat swab | exacerbation |
| 48 | 10653207 | 0.999973 | 57 | 15 | male | nasal swab | exacerbation |
| 49 | 10653207 | 0.999982 | 55 | 15 | male | Sputum | exacerbation |
| 60 | 6455190 | 0.999983 | 61 | 19 | female | Sputum | routine |
| 63 | 15110236 | 0.999985 | 61 | 6 | male | throat swab | routine |
| 64 | 15110236 | 0.999984 | 58 | 6 | male | nasal swab | routine |
| 65 | 15110236 | 0.999993 | 60 | 6 | male | Sputum | routine |
| 66 | 4047176 | 0.999985 | 58 | 22 | female | throat swab | routine |
| 67 | 4047176 | 1 | 56 | 22 | female | nasal swab | routine |
| 68 | 4047176 | 0.999983 | 59 | 22 | female | Sputum | routine |
| 72 | 14338705 | 0.999991 | 58 | 17 | male | nasal swab | end of antibiotic treatment |
| 73 | 14338705 | 0.999986 | 57 | 17 | male | Sputum | end of antibiotic treatment |
| 76 | 9765735 | 0.999986 | 58 | 16 | female | throat swab | end of antibiotic treatment |
| 77 | 9765735 | 0.999985 | 60 | 16 | female | nasal swab | end of antibiotic treatment |
| 78 | 9765735 | 0.999993 | 62 | 16 | female | Sputum | end of antibiotic treatment |
| 79 | 8985839 | 0.999985 | 59 | 16 | male | throat swab | exacerbation |
| 80 | 8985839 | 0.999975 | 57 | 16 | male | nasal swab | exacerbation |
| 81 | 8985839 | 0.999977 | 57 | 16 | male | Sputum | exacerbation |
| 93 | 10653207 | 1 | 59 | 15 | male | throat swab | end of antibiotic treatment |
| 94 | 10653207 | 0.999984 | 61 | 15 | male | nasal swab | end of antibiotic treatment |
| 95 | 10653207 | 0.999984 | 62 | 15 | male | Sputum | end of antibiotic treatment |
| 97 | 13029601 | 0.999992 | 56 | 18 | female | throat swab | exacerbation |
| 98 | 13029601 | 0.99996 | 57 | 18 | female | nasal swab | exacerbation |
| 99 | 13029601 | 0.999982 | 52 | 18 | female | Sputum | exacerbation |
| 100 | 19180069 | 0.999968 | 56 | 12 | female | nasal swab | routine |
| 101 | 19180069 | 0.999969 | 57 | 12 | female | Sputum | routine |
| 118 | 19254720 | 0.999978 | 54 | 13 | female | throat swab | routine |
| 119 | 19254720 | 0.999965 | 53 | 13 | female | nasal swab | routine |
| 120 | 19254720 | 0.999985 | 50 | 13 | female | Sputum | routine |
| 121 | 16346365 | 0.999985 | 56 | 8 | female | throat swab | routine |
| 122 | 16346365 | 1 | 55 | 8 | female | nasal swab | routine |
| 123 | 16346365 | 0.999992 | 55 | 8 | female | Sputum | routine |
| 128 | 11412475 | 0.999991 | 58 | 18 | male | throat swab | routine |
| 129 | 11412475 | 0.999979 | 60 | 18 | male | nasal swab | routine |
| 130 | 11412475 | 1 | 60 | 18 | male | Sputum | routine |
| 131 | 13029601 | 0.999978 | 59 | 18 | female | throat swab | end of antibiotic treatment |
| 132 | 13029601 | 0.999978 | 56 | 18 | female | nasal swab | end of antibiotic treatment |
| 133 | 13029601 | 0.999979 | 56 | 18 | female | Sputum | end of antibiotic treatment |
| 139 | 18709406 | 0.999984 | 58 | 20 | female | Sputum | routine |
| 142 | 14401148 | 0.999992 | 57 | 15 | female | throat swab | exacerbation |
| 143 | 14401148 | 0.999934 | 62 | 15 | female | nasal swab | exacerbation |
| 144 | 14401148 | 0.999985 | 58 | 15 | female | Sputum | exacerbation |
| 158 | 9765735 | 0.999993 | 56 | 16 | female | Sputum | routine |
| 168 | 20169885 | 1 | 58 | 13 | male | throat swab | routine |
| 169 | 20169885 | 0.999939 | 63 | 13 | male | nasal swab | routine |
| 170 | 20169885 | 0.999984 | 57 | 13 | male | Sputum | routine |
| 177 | 13029601 | 0.999984 | 58 | 18 | female | throat swab | routine |
| 178 | 13029601 | 0.99992 | 58 | 18 | female | nasal swab | routine |
| 179 | 13029601 | 0.999991 | 58 | 18 | female | Sputum | routine |
| 182 | 8173907 | 0.999992 | 58 | 17 | male | throat swab | exacerbation |
| 183 | 8173907 | 0.999991 | 60 | 17 | male | nasal swab | exacerbation |
| 184 | 8173907 | 0.999993 | 57 | 17 | male | Sputum | exacerbation |
| 191 | 14967982 | 0.999973 | 59 | 15 | female | throat swab | routine |
| 192 | 14967982 | 0.999973 | 62 | 15 | female | nasal swab | routine |
| 193 | 14967982 | 0.999985 | 61 | 15 | female | Sputum | routine |
| 198 | 18709406 | 0.999977 | 58 | 20 | female | throat swab | exacerbation |
| 199 | 18709406 | 0.999944 | 61 | 20 | female | nasal swab | exacerbation |
| 200 | 18709406 | 1 | 59 | 20 | female | Sputum | exacerbation |
| 206 | 13029601 | 0.999951 | 62 | 18 | female | BAL | exacerbation |
| 207 | 19254720 | 0.999977 | 57 | 13 | female | throat swab | routine |
| 208 | 19254720 | 1 | 61 | 13 | female | nasal swab | routine |
| 209 | 19254720 | 1 | 59 | 13 | female | Sputum | routine |
| 219 | 16346365 | 0.999985 | 60 | 8 | female | throat swab | routine |
| 220 | 16346365 | 0.999966 | 58 | 8 | female | nasal swab | routine |
| 221 | 16346365 | 0.999984 | 61 | 8 | female | Sputum | routine |
| 222 | 20169885 | 0.999985 | 59 | 13 | male | throat swab | routine |
| 223 | 20169885 | 0.999977 | 57 | 13 | male | nasal swab | routine |
| 224 | 20169885 | 0.999968 | 56 | 13 | male | Sputum | routine |
| 233 | 21856568 | 0.999971 | 57 | 13 | female | throat swab | end of antibiotic treatment |
| 234 | 21856568 | 0.999907 | 58 | 13 | female | nasal swab | end of antibiotic treatment |
| 235 | 21856568 | 0.999985 | 58 | 13 | female | Sputum | end of antibiotic treatment |
| 246 | 13029601 | 0.999993 | 59 | 18 | female | Sputum | end of antibiotic treatment |
| 249 | 6455190 | 0.999985 | 62 | 19 | female | throat swab | exacerbation |
| 250 | 6455190 | 0.999962 | 56 | 19 | female | nasal swab | exacerbation |
| 251 | 6455190 | 0.999977 | 58 | 19 | female | Sputum | exacerbation |
| 256 | 10653207 | 0.999966 | 57 | 16 | male | throat swab | exacerbation |
| 257 | 10653207 | 0.999983 | 61 | 16 | male | nasal swab | exacerbation |
| 258 | 10653207 | 1 | 58 | 16 | male | Sputum | exacerbation |
| 271 | 13568774 | 0.999971 | 57 | 20 | male | throat swab | exacerbation |
| 272 | 13568774 | 0.999934 | 55 | 20 | male | nasal swab | exacerbation |
| 273 | 13568774 | 1 | 59 | 20 | male | Sputum | exacerbation |
