# Supplementary material for: Comparison of Microbiomes from Different Niches of Upper and Lower Airways in Children and Adolescents with Cystic Fibrosis
Source: PLoS One. 2015 Jan 28;10(1):e0116029. doi: 10.1371/journal.pone.0116029 (PMC4309611; doi:10.1371/journal.pone.0116029)
Supplement: S2 Table — (DOCX) [file pone.0116029.s004.docx]

**Supplementary Table 2.** *Summary of CFTR genotypes with pancreatic status of patients with cystic fibrosis*

| Pancreatic insufficient | | Pancreatic sufficient | |
| --- | --- | --- | --- |
| *CFTR* Genotypes | **number of subjects n** | ***CFTR* Genotypes** | **number of subjects n** |
|  |  |  |  |
|  |  |  |  |
| F508del / F508del | 9 | F508del / R347P | 1 |
| F508del / 1341+1G>A | 1 | F508del / G551D | 1 |
| F508del / 2183AA>G | 1 | N1303K / L997del | 1 |
| F508del / 3905insT | 1 |  |  |
| F508del / 621+1G>T | 1 |  |  |
| F508del / N1303K | 1 |  |  |
| F508del / V520F | 1 |  |  |
| N1303k / N1303k | 1 |  |  |
| R1162X / Q525X | 1 |  |  |
